# High Glucose Induces Endothelial COX2 and iNOS Expression via Inhibition of Monomethyltransferase SETD8 Expression

**DOI:** 10.1155/2020/2308520

**Published:** 2020-02-23

**Authors:** Jie Qi, Qichao Wu, Qian Cheng, Xiangyuan Chen, Minmin Zhu, Changhong Miao

**Affiliations:** Department of Anaesthesiology, Fudan University Shanghai Cancer Center, Department of Oncology, Shanghai Medical College, Fudan University, Shanghai 200032, China

## Abstract

Cyclooxygenase 2 (COX2) and inducible nitric oxide synthase (iNOS) overexpression results in endothelial apoptosis, thus mediating vascular endothelial injury in hyperglycaemia. E26 transformation-specific sequence transcription factor-1 (ESE-1), which belongs to the E26 transformation-specific family of transcription factors, has been demonstrated to be involved in COX2 and iNOS gene transcription. Our previous study indicated that SET domain-containing protein 8 (SETD8) downregulation is involved in high glucose-mediated endothelial inflammation in human umbilical vein endothelial cells (HUVECs). Here, we report that SETD8 plays a major role in hyperglycaemia-induced COX2 and iNOS expression. In HUVECs, upregulation of ESE-1 expression was related to high glucose-mediated apoptosis and COX2 and iNOS expression. High glucose inhibited SETD8 expression, and overexpression of SETD8 diminished the effects of high glucose treatment. Consistently, RNA silencing of SETD8 led to the opposite effect. Furthermore, SETD8 was found to interact with specificity protein 1 (SP1). Blockade of SP1 protected against high glucose-mediated endothelial injury. Mechanistically, we showed that H4K20me1, a downstream target of SETD8, and SP1 were enriched at the ESE-1 promoter region by ChIP assay. Luciferase reporter assays indicated that SETD8 overexpression attenuated ESE-1 promoter activity and augmented the inhibitory effect of siSP1 on ESE-1 promoter activity. In general, our data indicate that SETD8 interacts with SP1 to coregulate ESE-1 expression, which is involved in hyperglycaemia-mediated endothelial apoptosis in HUVECs.

## 1. Introduction

The prevalence of diabetes has been increasing dramatically for decades, resulting in a growing public health problem worldwide [1]. The morbidity and mortality rates of diabetic patients with cardiovascular complications are 2- to 8-fold higher than those in nondiabetic patients [2, 3]. Accumulating evidence suggests that endothelial apoptosis is involved in the pathogenesis of cardiovascular complications in diabetic patients [4–6]. Proinflammatory enzymes, including cyclooxygenase 2 (COX2) and inducible nitric oxide synthase (iNOS), have been reported to participate in hyperglycaemia-mediated endothelial apoptosis and the occurrence of diabetes-related complications [7, 8]. Moreover, inhibition of COX2 and/or iNOS protects against high glucose-mediated endothelial apoptosis [7, 8].

E26 transformation-specific sequence (ETS) transcription factor-1 (ESE-1) was originally identified as an epithelial-restricted ETS factor [9]. ESE-1 expression is increased upon inflammatory stimuli in vascular endothelial cells [10]. Moreover, ESE-1 has been reported to be involved in the activation of COX2 [11] and iNOS [10] gene expression by modulating transcription, thus participating in endothelial apoptosis. As an inducer of COX2 and iNOS gene expression [10, 11], ESE-1 may become a potential target for hyperglycaemia-mediated endothelial apoptosis and injury.

SET domain-containing protein 8 (SETD8), also known as SET8, is the only known lysine methyltransferase responsible for specific monomethylation on histone H4 at lysine 20 (H4K20me1) [12]. The methyltransferase activity of SETD8 is involved in a variety of crucial cellular functions, including DNA repair, cell cycle progression, transcriptional and posttranslational regulation, and cellular metabolism [12, 13]. We previously indicated that SETD8 downregulation is involved in high glucose-mediated endothelial inflammation in HUVECs [14]. In the present study, we hypothesize that SETD8 downregulation may increase ESE-1 expression, playing a crucial role in hyperglycaemia-induced endothelial apoptosis in HUVECs. More importantly, we also examined the potential mechanism by which SETD8 modulates ESE-1 expression.

## 2. Materials and Methods

### 2.1. Cell Culture and Reagent

Human umbilical vein endothelial cells (HUVECs) were obtained from American Type Culture Collection (ATCC; Manassas, USA) and cultured in Dulbecco's modified Eagle medium (DMEM) with 5 mM glucose containing 1% penicillin-streptomycin and 10% foetal bovine serum at 37°C in a humidified 5% carbon dioxide incubator.

For high glucose treatment, cells were washed with PBS to remove the complete medium and further cultured in DMEM supplemented with high glucose (25 mM) for 3 days. Glucose (5 mM) plus mannitol (20 mM) was used as an osmotic control.

### 2.2. Apoptosis Assay

Apoptosis was measured by fluorescence-activated cell sorting (FACS) analysis (Cytomics FC 500 MPL; Beckman Coulter, Fullerton, USA) using double staining with annexin V-FITC and propidium iodide (PI; BD Biosciences, San Jose, USA). Briefly, after different treatments, cells were harvested and incubated with PI and annexin V-FITC for 30 min at 37°C in the dark and then analysed by flow cytometry.

### 2.3. Western Blot Analysis

Cell extracts were prepared by using Cell Lysis Buffer (Cell Signaling Technology, Danvers, USA). Protein samples were boiled in sample loading buffer for 5 minutes, and equal amounts of proteins from different groups of HUVECs were separated by 10% SDS-PAGE and transferred to PVDF membranes (Millipore, Billerica, USA). Membranes were blocked with 5% fat-free milk solution for 1 h, and then, the membranes were incubated with primary antibodies overnight at 4°C. The primary antibodies used were monoclonal antibodies against *β*-actin (ProteinTech, 1/5000), SETD8 (ProteinTech, 1/1000), H4K20me1 (Abcam, 1/1000), SP1 (ProteinTech, 1/1000), ESE-1 (R&D, 1/1000), iNOS (Affinity antibody, 1/1000), and COX2 (Affinity antibody, 1/1000). After washing the membranes with Tris-buffered saline containing Tween-20, the corresponding HRP-conjugated secondary antibody was added at room temperature for 1 hour, and the membrane was further washed with TBS-T 5 times. Subsequently, the signal was detected by the ECL system. The density of the protein bands was analysed by Scan-gel-it software. Protein expression was normalized to *β*-actin expression.

### 2.4. Quantitative PCR

Total RNA was isolated by TRIzol® reagent (Tiangen Biotech, Beijing, China). cDNA was synthesized using Hifair® II 1st Strand cDNA Synthesis SuperMix for qPCR (gDNA digester plus) (Yeasen, Shanghai, China). Quantitative real-time PCR (qPCR) was performed by using Hieff UNICON® qPCR TaqMan Probe Master Mix (Yeasen, Shanghai, China) to analyse the gene expression of *β*-actin, SETD8, SP1, ELF3, iNOS, and COX2 with the QuantStudio 7 Flex Real-Time PCR System (Applied Biosystems, Waltham, USA). The qPCR primer sequences used in this study can be found in Table 1.

### 2.5. Coimmunoprecipitation (Co-IP)

Whole cell protein lysates were extracted with a cell lysis buffer containing PMSF (Beyotime Biotechnology, Shanghai), and 30 *μ*l of each lysate was used as input. For endogenous IP, lysates were incubated with the corresponding primary antibodies and 50 *μ*l protein A/G Dynabeads (Thermo Fisher, USA) at 4°C overnight. Then, 10 *μ*l of input and IP were subjected to Western blotting.

### 2.6. Chromatin Immunoprecipitation (ChIP) Assay

ChIP assays were carried out with a Simple ChIP Plus Sonication Chromatin IP Kit (Cell Signaling Technology, MA) according to the manufacturer's instructions. Briefly, cells (1 × 10^7^) were fixed with 1% formaldehyde for 10 min at room temperature to cross-link DNA and proteins. Glycine was then added to stop the cross-linking reaction. Chromatin was sheared using a Microson Ultrasonic Cell Disruptor XL (Misonix) with 16 cycles of sonication (15 s each, 2 min rest, amplitude = 10, power = 15 W). Ten microliters of sonicate was collected from each sample as an input control, while the remaining sample was incubated with anti-SP1 (Abcam, USA), anti-H4K20me1 (Abcam, USA) antibodies, histone H3-positive control, or IgG negative control at 4°C overnight. Immunoprecipitants were bound to protein G magnetic beads, and the DNA-protein cross-link was reversed at 65°C for 2 h. DNA was purified, and enriched DNA sequences were analysed by qPCR. ESE-1 oligonucleotide sequences for PCR primers were forward 5′-TGCAATTGTGCCCTTGAGGA-3′ and reverse 5′-CCTACGGCCACACTGAACTC-3′.

### 2.7. Dual-Luciferase Assay

The Promega Dual-Luciferase Assay Kit (Madison, WI, United States) was used to assess the impact of SETD8 and SP1 on ESE-1 promoter activity. The ESE-1 promoter was amplified from genomic DNA of 293T cells and ligated into the pGL3-Basic vector to generate the pGL3-ELF3 construct. pGL3-ELF3 was transfected with a Renilla luciferase vector, and the impact of SETD8 and SP1 on ESE-1 promoter activity was assessed using the Dual-Luciferase Assay Kit.

### 2.8. Small Interfering RNA (siRNA) and Short Hairpin RNA (shRNA) Treatments

HUVECs were transfected with shRNA against SETD8 and siRNA against SP1 and ESE-1 using Lipofectamine 3000 (Invitrogen, USA) according to the manufacturer's instructions.

The sequences of shSETD8 (Biotend, Shanghai) were as follows: shSETD8a, 5′-CAACAGAATCGCAAACTTA-3′, shSETD8b, 5′-CAACAGAATCGCAAACTTA-3′. The sequences of siSP1a siRNA (Biotend, Shanghai) were as follows: siSP1a, sense, 5′-CAUCCAAGGCUGUGGGAAAdTdT-3′, anti-sense, 5′-UUUCCCACAGCCUUGGAUGdTdT-3′; siSP1b, sense, 5′-GCACAAACGUACACACACAdTdT-3′, anti-sense, 5′-UGUGUGUGUACGUUUGUGCdTdT-3′.

The sequences of ESE-1 siRNAs (Biotend, Shanghai) were as follows: siESE-1a, sense, 5′-GCCAUUGACUUCUCACGAUdTdT-3′, anti-sense, 5′-AUCGUGAGAAGUCAAUGGCdTdT-3′; siESE-1b, sense, 5′-GCCAUGAGGUACUACUACAdTdT-3′, anti-sense, 5′-UGUAGUAGUACCUCAUGGCdTdT-3′.

### 2.9. Statistical Analysis

The results are presented as the mean ± SD (standard deviation) from five separately performed experiments. Two-tailed unpaired *t*-tests or one-way ANOVA with GraphPad Prism Version 6 (GraphPad Software, San Diego, CA) was performed to compare the groups. *P* < 0.05 was considered statistically significant.

## 3. Results

### 3.1. High Glucose Induced COX2 and iNOS Expression via Upregulation of ESE-1 in HUVECs

**Figure 1:**
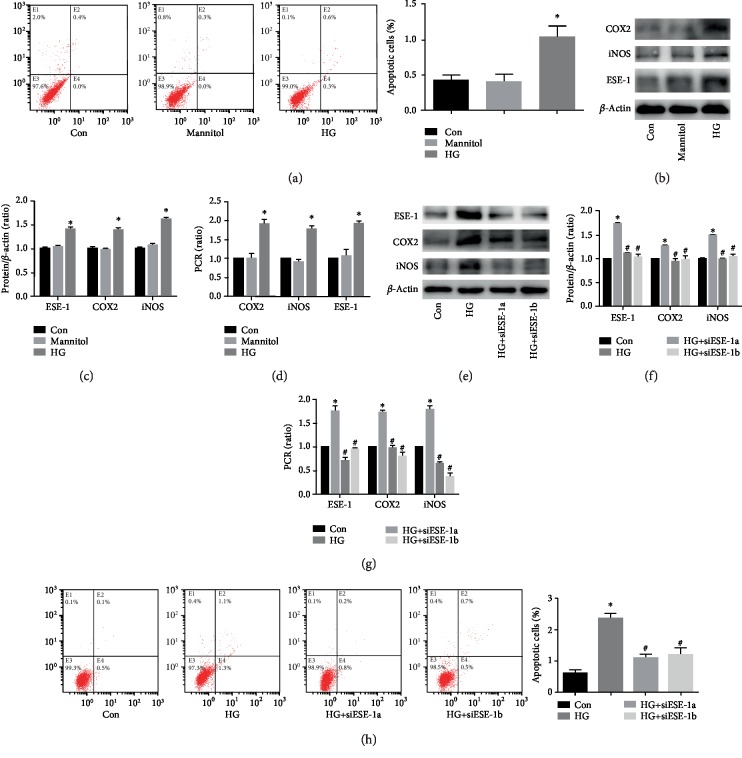
High glucose induced cyclooxygenase 2 (COX2) and inducible nitric oxide synthase (iNOS) expression via upregulation of E26 transformation-specific sequence transcription factor-1 (ESE-1) in human umbilical vein endothelial cells (HUVECs). (a) Flow cytometry was used to detect apoptotic HUVECs cultured in normal glucose, osmotic control, or high glucose medium. Compared with the control group, high glucose treatment increased apoptosis in HUVECs. Osmotic control had no effect on cell apoptosis. (b, c) Western blot analysis of ESE-1, iNOS, and COX2 in HUVECs cultured in normal glucose, osmotic control, or high glucose conditions. Compared with the control group, high glucose treatment increased ESE-1, iNOS, and COX2 protein expression in HUVECs. The osmotic control had no effect on cell apoptosis. (d) The mRNA expression of ESE-1, iNOS, and COX2 was examined by qPCR in HUVECs grown in normal glucose medium, osmotic control medium, or high glucose medium. Compared with the control group, high glucose treatment increased ESE-1, iNOS, and COX2 mRNA expression in HUVECs. The osmotic control had no effect on cell apoptosis. (e, f) The effects of siESE-1 on high glucose-induced COX2 and iNOS expression were measured by Western blot analysis in HUVECs. siESE-1 decreased ESE-1, iNOS, and COX2 protein expression in hyperglycaemic HUVECs. (g) The effects of siESE-1 on high glucose-induced COX2 and iNOS expression were measured by qPCR in HUVECs. siESE-1 decreased ESE-1, iNOS, and COX2 mRNA expression in hyperglycaemic HUVECs. (h) The effects of siESE-1 on high glucose-induced apoptosis in HUVECs. siESE-1 decreased cell apoptosis in hyperglycaemic HUVECs. siESE-1: small interfering RNA ESE-1. ^∗^*P* < 0.05, compared with the control group; ^#^*P* < 0.05, compared with high glucose treatment.

COX2 and iNOS overexpression results in endothelial apoptosis, thus mediating vascular endothelial injury in hyperglycaemia [7, 8]. To determine whether high glucose could mediate endothelial apoptosis in HUVECs, cells were subincubated in different types of media: normal glucose (Con, 5 mM, 3 days), osmotic control (mannitol, 5 mM glucose plus 20 mM mannitol, 3 days), and high glucose (HG, 25 mM, 3 days). The results indicated that high glucose induced cell apoptosis (Figure 1(a)) and increased the expression of COX2 and iNOS at the protein (Figures 1(b) and 1(c)) and mRNA (Figure 1(d)) levels in HUVECs. Previous studies have shown that ESE-1 activates the expression of COX2 [11] and iNOS [10], so we detected ESE-1 expression under high glucose treatment conditions. Accordingly, we observed that high glucose treatment augmented ESE-1 expression (Figures 1(b)–1(d)). To further clarify the role of ESE-1 in regulating COX2 and iNOS expression in hyperglycaemic HUVECs, we used two independent siRNAs against ESE-1. The effects of siESE-1 were validated by Western blotting (Figures 1(e) and 1(f)) and quantitative real-time PCR (Figure 1(g)). The results showed that silencing ESE-1 decreased high glucose-induced COX2 and iNOS expression (Figures 1(e)–1(g)) and rescued cell apoptosis (Figure 1(h)) induced by high glucose in HUVECs. These data indicated that ESE-1 positively regulates COX2 and iNOS expression, thus mediating apoptosis in hyperglycaemic HUVECs.

### 3.2. SETD8 Downregulation Participated in High Glucose-Induced COX2 and iNOS Expression via Increasing ESE-1 Expression in HUVECs

**Figure 2:**
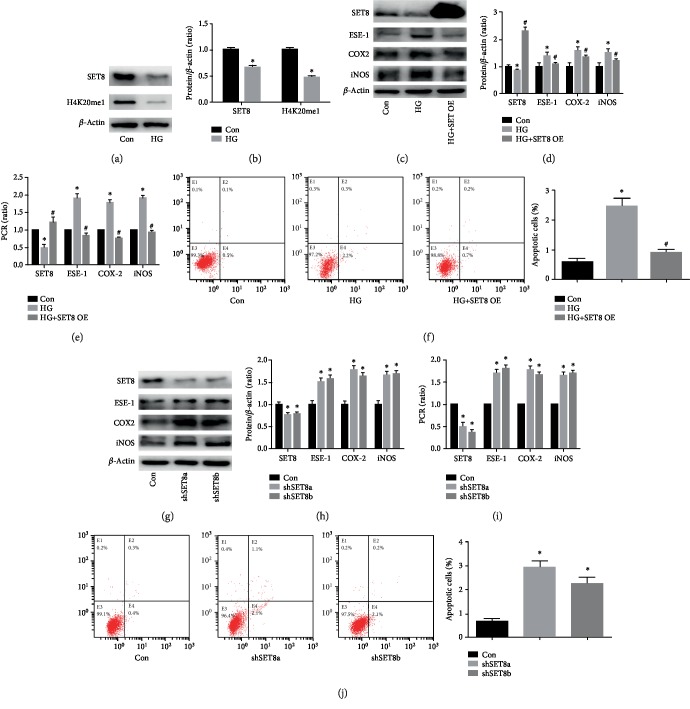
SET domain-containing protein 8 (SETD8) overexpression counteracted high glucose-induced cyclooxygenase 2 (COX2) and inducible nitric oxide synthase (iNOS) expression in human umbilical vein endothelial cells (HUVECs). (a, b) Western blot analysis of SETD8 and H4K20me1 in HUVECs cultured under normal or high glucose conditions. Compared with the control group, high glucose treatment decreased SETD8 and H4K20me1 protein expression in HUVECs. (c, d) Western blot analysis of SETD8, E26 transformation-specific sequence transcription factor-1 (ESE-1), iNOS, and COX2 in HUVECs overexpressing SETD8 under high glucose conditions. SETD8 overexpression inhibited ESE-1, iNOS, and COX2 protein expression in hyperglycaemic HUVECs. (e) qPCR analysis of SETD8, ESE-1, iNOS, and COX2 in HUVECs overexpressing SETD8 under high glucose conditions. SETD8 overexpression inhibited ESE-1, iNOS, and COX2 mRNA expression in hyperglycaemic HUVECs. (f) The effects of SETD8 overexpression on high glucose-mediated apoptosis in HUVECs. SETD8 overexpression decreased cell apoptosis in hyperglycaemic HUVECs. (g, h) Western blot analysis of SETD8, ESE-1, iNOS, and COX2 in HUVECs upon SETD8 silencing. Compared with the control group, shSETD8 increased ESE-1, iNOS, and COX2 protein expression in HUVECs. (i) qPCR analysis of SETD8, ESE-1, iNOS, and COX2 in HUVECs upon SETD8 silencing. Compared with the control group, shSETD8 increased ESE-1, iNOS, and COX2 mRNA expression in HUVECs. (j) Cell apoptosis analysis in HUVECs upon SETD8 silencing. Compared with the control group, shSETD8 increased cell apoptosis in HUVECs. SETD8 OE: SETD8 overexpression; shSETD8: short hairpin RNA SETD8. ^∗^*P* < 0.05, compared with the control group; ^#^*P* < 0.05, compared with high glucose treatment.

**Figure 3:**
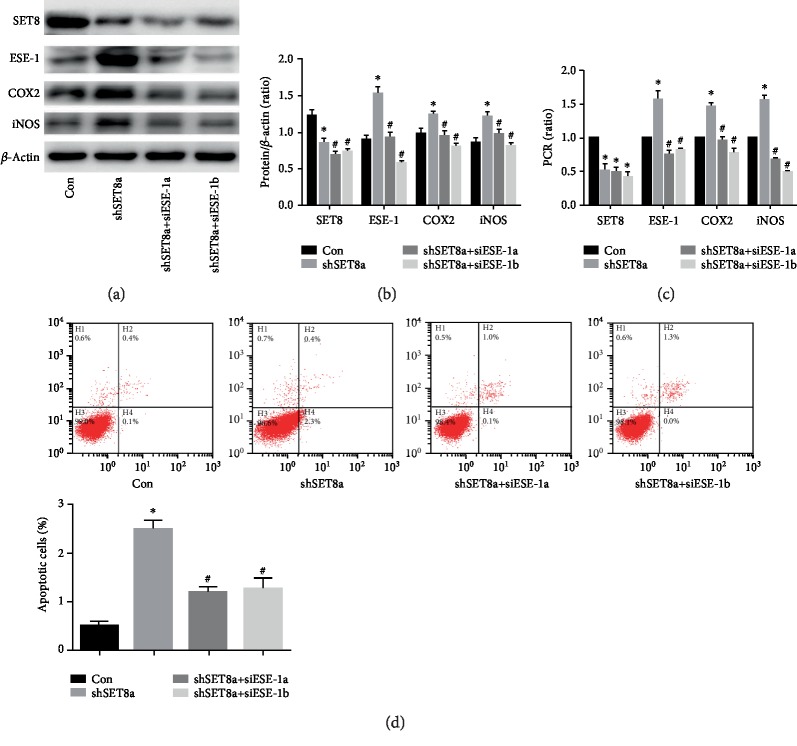
SET domain-containing protein 8 (SETD8) downregulation induced cyclooxygenase 2 (COX2) and inducible nitric oxide synthase (iNOS) expression via upregulation of E26 transformation-specific sequence transcription factor-1 (ESE-1) in human umbilical vein endothelial cells (HUVECs). (a, b) Western blot analysis of SETD8, ESE-1, iNOS, and COX2 expression in HUVECs with SETD8 and ESE-1 knockdown. siESE-1 inhibited shSETD8-induced iNOS and COX2 protein expression in HUVECs. (c) qPCR analysis of SETD8, ESE-1, iNOS, and COX2 expression in HUVECs with SETD8 and ESE-1 knockdown. siESE-1 inhibited shSETD8-induced iNOS and COX2 mRNA expression in HUVECs. (d) Cell apoptosis analysis in HUVECs with SETD8 and ESE-1 knockdown. siESE-1 alleviated shSETD8-mediated cell apoptosis in HUVECs. shSETD8: short hairpin RNA SETD8; siESE-1: small interfering RNA ESE-1. ^∗^*P* < 0.05, compared with the control group; ^#^*P* < 0.05, compared with high glucose treatment.

We previously indicated that SETD8 downregulation participates in high glucose-mediated endothelial injury in HUVECs [14]. Consistently, SETD8 was found to be downregulated by high glucose treatment in the present study (Figures 2(a) and 2(b)). H4K20me1, a downstream target of SETD8, was also significantly decreased by high glucose treatment (Figures 2(a) and 2(b)). To investigate the effect of SETD8 in high glucose-mediated HUVEC injury, both loss- and gain-of-function approaches were employed. Our data indicated that SETD8 overexpression counteracted high glucose-induced ESE-1, COX2, and iNOS expression (Figures 2(c)–2(e)) and attenuated high glucose-mediated cell apoptosis (Figure 2(f)). Moreover, the effects of shSETD8 were similar to those of high glucose treatment (Figures 2(g)–2(j)). To explore whether the effects of shSETD8 were achieved via upregulation of ESE-1 expression, we silenced ESE-1 in SETD8 knockdown HUVECs. Downregulation of ESE-1 reversed SETD8 silencing-induced COX2 and iNOS expression (Figures 3(a)–3(c)) and apoptosis (Figure 3(d)). These data indicated that SETD8 downregulation led to the upregulation of ESE-1 in hyperglycaemic HUVECs, thus mediating COX2 and iNOS expression, cell viability, and apoptosis.

### 3.3. SETD8 Interacted with SP1

**Figure 4:**
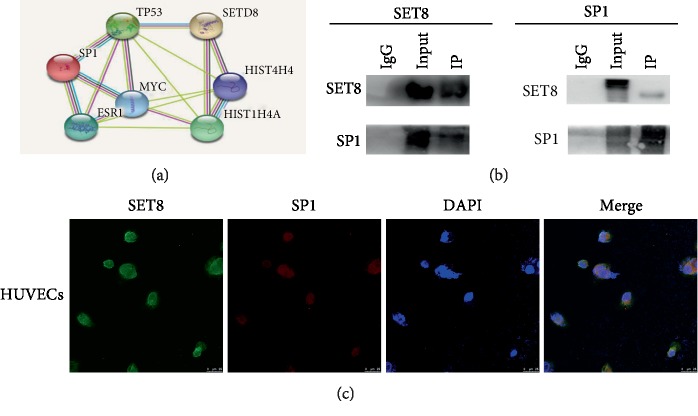
SET domain-containing protein 8 (SETD8) interacted with specificity protein 1 (SP1). (a) Several transcription factors that interact with SETD8 (https://string-db.org). (b) The interaction between SETD8 and SP1 in HUVECs was measured by immunoprecipitation. SETD8 was detected to interact with SP1 in HUVECs by Co-IP. (c) Colocalization of SETD8 and SP1 in HUVECs as detected by confocal microscopy.

Next, to search for a possible regulatory mechanism, we predicted the proteins that interact with SETD8 using bioinformatics. Several transcription factors are shown in Figure 4(a) (https://string-db.org). Coimmunoprecipitation assays verified that SETD8 interacted with SP1 in HUVECs (Figure 4(b)). Moreover, immunofluorescence assays showed that SETD8 and SP1 were colocalized in the nucleus (Figure 4(c)).

### 3.4. SP1 Upregulation Participated in High Glucose-Induced COX2 and iNOS Expression via an Increase in ESE-1 Expression in HUVECs

**Figure 5:**
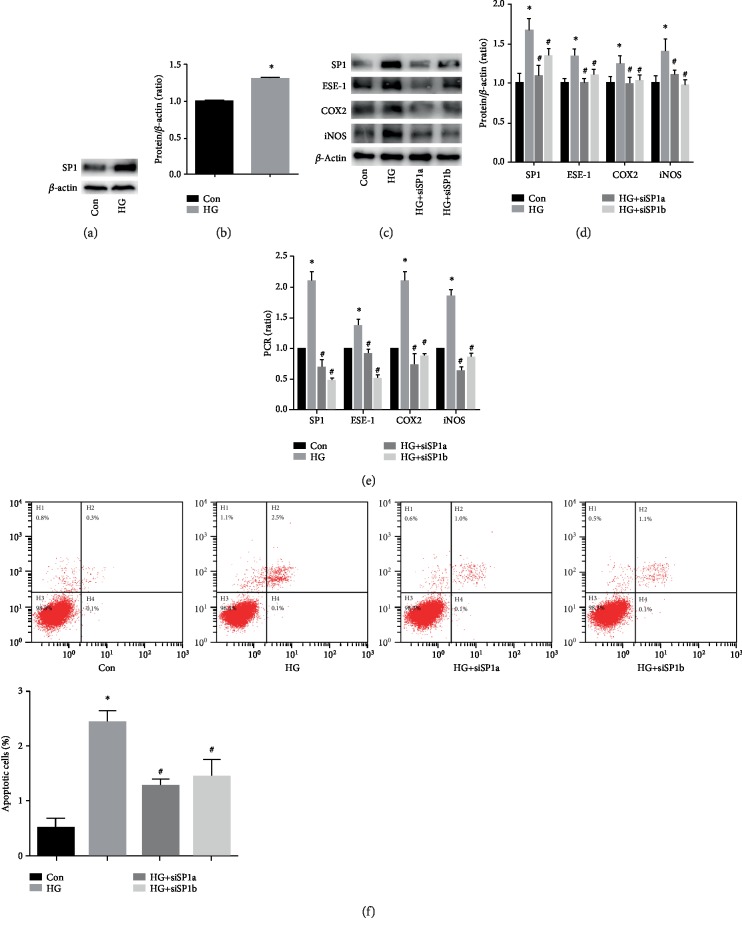
Specificity protein 1 (SP1) downregulation counteracted high glucose-induced E26 transformation-specific sequence transcription factor-1 (ESE-1) expression and endothelial apoptosis. (a, b) Western blot analysis of SP1 in HUVECs cultured under normal or high glucose conditions. Compared with the control group, high glucose treatment increased SP1 protein expression in HUVECs. (c, d) Western blot analysis of SP1, ESE-1, cyclooxygenase 2 (COX2), and inducible nitric oxide synthase (iNOS) in HUVECs with siSP1 under high glucose conditions. siSP1 decreased ESE-1, iNOS, and COX2 protein expression in hyperglycaemic HUVECs. (e) qPCR analysis of SP1, ESE-1, iNOS, and COX2 in HUVECs with siSP1 under high glucose conditions. siSP1 decreased ESE-1, iNOS, and COX2 mRNA expression in hyperglycaemic HUVECs. (f) The effects of siSP1 on high glucose-mediated apoptosis in HUVECs. siSP1 decreased cell apoptosis in hyperglycaemic HUVECs. siSP1: small interfering RNA SP1. ^∗^*P* < 0.05, compared with the control group; ^#^*P* < 0.05, compared with high glucose treatment.

In the present study, SP1 was found to be upregulated by high glucose treatment (Figures 5(a) and 5(b)). Moreover, SP1 downregulation counteracted high glucose-induced ESE-1, COX2, and iNOS expression (Figures 5(c)–5(e)) and reversed high glucose-mediated cell apoptosis (Figure 5(f)). These data indicated that SP1 downregulation inhibited ESE-1 expression in hyperglycaemic HUVECs, thus inhibiting COX2 and iNOS expression and attenuating apoptosis.

### 3.5. SETD8 Interacted with SP1 to Regulate ESE-1 Promoter Activity in HUVECs

**Figure 6:**
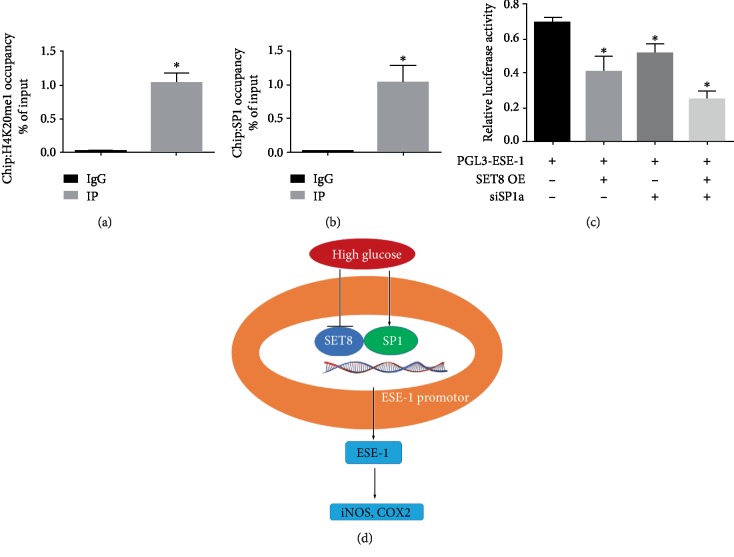
SET domain-containing protein 8 (SETD8) interacts with specificity protein 1 (SP1) to regulate E26 transformation-specific sequence transcription factor-1 (ESE-1) expression in hyperglycaemic HUVECs. (a) H4K20me1 is enriched at the ESE-1 promoter region. (b) SP1 is enriched at the ESE-1 promoter region. (c) Luciferase reporter assays indicated that SETD8 overexpression not only attenuated ESE-1 promoter activity but also augmented the effect of siSP1a on ESE-1 promoter activity. (d) Schematic representation of the working model. SETD8 OE: SETD8 overexpression; siSP1: small interfering RNA SP1. ^∗^*P* < 0.05, compared with the control group.

To further identify whether ESE-1 is targeted by SETD8 and SP1, we examined the genome-wide distribution of H4K20me1, a downstream target of SETD8, and SP1 by ChIP assay in HUVECs. Our data indicated that both H4K20me1 (Figure 6(a)) and SP1 (Figure 6(b)) were enriched in the ESE-1 promoter region. Moreover, luciferase reporter assays showed that SETD8 overexpression not only attenuated ESE-1 promoter activity but also augmented the effect of siSP1a on ESE-1 promoter activity (Figure 6(c)). These data demonstrated that SETD8 interacted with SP1 to regulate ESE-1 promoter activity in hyperglycaemic HUVECs, thus facilitating high glucose-induced COX2 and iNOS expression (Figure 6(d)).

## 4. Discussion

The main finding of this study is that high glucose, via upregulation of ESE-1 expression, induced COX2 and iNOS expression, leading to endothelial cell apoptosis. Moreover, high glucose decreased SETD8 expression and increased SP1 expression. In addition, ChIP assays revealed that H4K20me1 and SP1 were enriched at the promoter region of ESE-1. Further investigation demonstrated that SETD8 interacted with the transcription factor SP1 to coregulate ESE-1 transcription activity in hyperglycaemic HUVECs.

Hyperglycaemia is crucial in vascular apoptosis [15], which participates in the pathogenesis of cardiovascular complications in diabetic patients [4–6]. The inflammatory factor COX2 is involved in hyperglycaemia-mediated endothelial apoptosis and the occurrence of diabetes-related complications [7], including diabetic retinopathy [16], vascular leakage [17], and abnormalities [18]. Similarly, iNOS also contributed to apoptosis [8, 19] and dysfunction [20] in hyperglycaemic endothelial cells. Previous studies have indicated that ESE-1 participates in endothelial inflammatory processes via activation of COX2 [11] and iNOS [10] gene transcription. Consistent with previous observations [10, 11], we found that ESE-1 silencing normalized high glucose-induced COX2 and iNOS expression and hyperglycaemia-induced cell apoptosis. These data indicated that ESE-1 may be a potential target for hyperglycaemia-mediated endothelial apoptosis and injury.

Our previous study showed that under hyperglycaemic conditions, SETD8 overexpression reduced the expression of adhesion molecules and therefore weakened high glucose-mediated endothelial inflammation [14]. Here, we observed similar results: SETD8 overexpression inhibited high glucose-induced ESE-1, COX2, and iNOS expression and reduced high glucose-induced cell apoptosis. To gain further insight into the mechanism underlying hyperglycaemia-induced apoptosis, we tested whether ESE-1 is a transcription target of SETD8. Our data showed that H4K20me1, a downstream target of SETD8, was enriched at the promoter region of ESE-1. Importantly, we found that ESE-1 downregulation counteracted the effect of SETD8 knockdown on high glucose-induced COX2 and iNOS expression. These data indicated that SETD8 overexpression may transcriptionally inhibit ESE-1 and thus reduce high glucose-induced COX2 and iNOS expression. Previous studies indicated that epigenetic changes were involved in the regulation of COX2 [21] and iNOS [22, 23] gene expression. Accordingly, we found that SETD8 epigenetically regulated COX2 and iNOS expression via modulation of ESE-1.

SP1 was previously reported to be responsible for high glucose-induced endothelial injury [24–26]. Other studies have demonstrated that high glucose induced plasminogen activator inhibitor-1 gene transcription via activation of SP1 transcription [24, 25]. Under hyperglycaemia, SP1 increased keap1 transcription, leading to endothelial oxidation and the occurrence of diabetic nephropathy [26]. Importantly, we found that SP1 was increased with high glucose treatment and SP1 was enriched at the promoter region of ESE-1. Moreover, we observed that ESE-1, COX2, and iNOS expression induced by high glucose was significantly reduced via inhibition of SP1. These data indicated that SP1 participated in high glucose-induced COX2 and iNOS expression via transcriptional regulation of ESE-1 expression.

It has been established that the transcriptional activity of SP1 can be regulated by epigenetic changes [26, 27]. In the present study, we demonstrated that SETD8 interacted with SP1. ChIP assays showed that both H4K20me1 and SP1 were occupied by the ESE-1 promoter region. Further, SETD8 overexpression augmented the effect of siSP1 on ESE-1 promoter activity. These data suggested that SETD8 interacts with SP1 to coregulate ESE-1 expression in hyperglycaemic HUVECs.

The study has some limitations. First, whether the interaction between SETD8 and SP1 is direct or indirect requires further research. Second, whether the SETD8/SP1 interaction or SETD8-mediated H4K20me1 modification counteracted the positive effect of SP1 on ESE-1 promoter activity deserves further clarification. Third, mechanistic studies carried out in HUVECs in the present study should be confirmed in *in vivo* studies.

## 5. Conclusion

In summary, the present study demonstrated that high glucose enhanced ESE-1, COX2, and iNOS expression, thus inducing endothelial apoptosis in HUVECs. Moreover, high glucose decreased SETD8 expression and increased SP1 expression. Mechanistically, SETD8 interacted with SP1 to coregulate ESE-1 expression, which participated in high glucose-mediated endothelial apoptosis in hyperglycaemic HUVECs.

## Figures and Tables

**Table 1:** Sequences of primers.

Genes	Sequence	
	Forward (5′-3′)	Reverse (5′-3′)
*β*-Actin	ATGCCCTGAGGCTCTTTTCCAGCC	CCAGGATGGAGCCACCGATCCACA
*SETD8*	AGCTCCAGGAAGAGCAAAGCCGAG	GGCGTCGGTGATCTCGATGAGGT
*SP1*	CGAAGTAGCAGCACAGGCAGTAG	GGAGCGGCAGCCACAACATAC
*ESE-1*	TGGAAGTGACGTGGACCTGGATC	GACGCCTTCATGCCGATTCTCC
*iNOS*	ACTACAGGCTCGTGCAGGACTC	CCACCACTCGCTCCAGGATACC
*COX2*	CCATTGACCAGAGCAGGCAGATG	TGGCTTCCAGTAGGCAGGAGAAC

## Data Availability

The data used in the present study to support the findings of this study are available from the corresponding author upon request.
